# Initial Feasibility and Acceptability of Cancer Pain 101: An Interdisciplinary, Single‐Session, Telehealth Intervention for Patients With Cancer‐Related Pain

**DOI:** 10.1002/cam4.70898

**Published:** 2025-05-02

**Authors:** Desiree R. Azizoddin, Jian Zhao, Sara M. DeForge, Meng Chen, Ryan Nipp, Jennifer Hardcopf Stagg, Kerry Bond, Raina Leckie, Blake T. Hilton, Jordan M. Neil, James A. Tulsky, William Pirl, Robert R. Edwards, Beth D. Darnall

**Affiliations:** ^1^ Health Promotion Research Center Stephenson Cancer Center, University of Oklahoma Health Sciences Center Oklahoma City Oklahoma USA; ^2^ Department of Psychosocial Oncology and Palliative Care Dana‐Farber Cancer Institute Boston Massachusetts USA; ^3^ Harvard Medical School Boston Massachusetts USA; ^4^ Department of Psychology University of Southern California Los Angeles California USA; ^5^ Section of Hematology/Oncology, Department of Medicine College of Medicine, University of Oklahoma Health Sciences Center Oklahoma City Oklahoma USA; ^6^ McLean Hospital Boston Massachusetts USA; ^7^ Department of Anesthesiology, Perioperative, and Pain Medicine Brigham and Women's Hospital Boston Massachusetts USA; ^8^ Department of Anesthesiology, Perioperative and Pain Medicine Stanford University School of Medicine Palo Alto California USA

**Keywords:** cancer pain, interdisciplinary, opioids, pain management, pain psychology, palliative care, telehealth

## Abstract

**Introduction:**

Patients with cancer have limited access to comprehensive pain treatment. We developed a 90‐min, single‐session, telehealth, interdisciplinary intervention that combines cancer‐specific medical education and behavioral pain treatment. We evaluated the intervention's preliminary feasibility and acceptability for patients with cancer‐related pain.

**Methods:**

Adults with cancer‐related pain (≥ 4/10, average pain) who are receiving cancer treatment (< 3 months) self‐enrolled or were recruited from the Stephenson Cancer Center (SCC) in Oklahoma. Patients completed a baseline survey and attended the 90‐min group‐based, Zoom‐delivered telehealth intervention. They completed post‐intervention, 2‐week, and 4‐week follow‐up assessments and an optional debriefing interview. The feasibility benchmark was ≥ 70% attendance and 80% of acceptability items rated ≥ 4/5.

**Results:**

Seventy participants (67.5% female; mean age = 52.5 years; 25% rural‐dwelling) enrolled. Forty of 70 (57%) attended the intervention. Of those, 95% completed the post‐intervention survey, and 90% and 95% completed the 2‐week and 4‐week follow‐ups, respectively. Participants reported high acceptability, understandability (97%), and relevance (90%). Most (80%) would recommend the class to others. Qualitative feedback highlighted reduced helplessness and fear regarding opioid use, adoption of behavioral pain management strategies, and appreciation for the convenience of telehealth. Exploratory analyses showed significant reductions in pain intensity (mean difference [MD] = 1.27, *p* = 0.001), pain interference (MD = 5.48, *p* = 0.017), pain catastrophizing (MD = 6.0, *p* = 0.003), sleep disturbance (MD = 3.64, *p* = 0.004), and depression (MD = 3.97, *p* = 0.018) at 4 weeks.

**Conclusion:**

While attendance was below feasibility benchmarks, this interdisciplinary, telehealth intervention was acceptable and improved self‐reported cancer pain management. Further research will identify barriers to improve attendance, and determine the optimal timing within the cancer trajectory to deliver pain self‐management content. Randomized controlled trials are needed to assess intervention efficacy on patient outcomes.

## Graphical abstract

To improve access to comprehensive pain management, an interdisciplinary team developed a 90‐min, single‐session, telehealth intervention that combines medical education with behavioral pain treatment modified for patients with cancer. In an open, single‐arm pilot study, the intervention was found to be moderately feasible, highly acceptable, and participants described reduced fear regarding opioid use, increased adoption of behavioral pain management strategies, and appreciation for the convenience of telehealth.

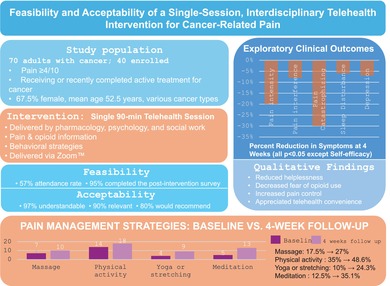

## 1 Introduction

Cancer pain is a common and distressing symptom that impacts patients' social, emotional, and physical functioning, and quality of life [1, 2, 3]. About 50%–60% of patients with cancer experience pain, with pain intensifying in more advanced stages [4]. Despite the availability of opioids to help with moderate to severe cancer pain, almost 60% of patients endure inadequate pain relief [5, 6]. Insufficient education about opioids and poor awareness of nonpharmacological pain coping strategies contribute to suboptimal pain management [7, 8]. While pharmacotherapy remains a critical component of cancer pain management, there is a clear need for comprehensive pain education that discusses safe and effective opioid use and nonpharmacological pain self‐management strategies while addressing the multifaceted nature of cancer pain [9, 10, 11].

Evidence supporting the efficacy of integrated, comprehensive treatment approaches for chronic cancer pain is widely recognized (e.g., pain‐cognitive behavioral therapy [12]) yet most patients with cancer receive unimodal pharmacologic pain treatment [13, 14]. Continued reliance on unimodal pain care may be partially explained by the limited availability of trained clinicians who offer comprehensive cancer pain treatment (e.g., palliative care, pain psychologists, and physical therapy), especially in rural and medically underserved areas [15, 16]. Single‐session or abbreviated interventions increase access to effective treatments using brief and accessible approaches [17, 18, 19, 20].

Telehealth has emerged as a potential strategy to bridge gaps in access to comprehensive pain treatment for individuals with pain [21, 22, 23]. Telephone and video‐based clinical appointments are viable alternatives to in‐person care [24] for pain self‐management and have demonstrated efficacy in patients with cancer pain [23, 25]. Existing interventions primarily focus on providing medication management assistance [25] and few incorporate behavioral pain self‐management elements that are integral to comprehensive pain care [26]. Despite the effectiveness of telehealth treatments, delivery of and patient adherence to multi‐session telehealth services are complicated by technological barriers and lengthy time commitments (e.g., eight, 60‐min video‐based sessions) [27, 28].

To address the limited accessibility of multimodal pain treatments, we developed a single‐session, telehealth intervention, which we call “Cancer Pain 101,” that integrates psychological and pharmacologic education specific to cancer pain. We sought to improve access to comprehensive cancer pain education for patients at the only NCI‐designated cancer center in Oklahoma; a state with over one‐third [29] of households being located in rural areas compared to one‐fifth of the national population [30]. Herein, we describe a single‐arm, open pilot study designed to evaluate the preliminary feasibility and acceptability of Cancer Pain 101 among patients experiencing cancer pain and explore the preliminary efficacy of the intervention on pain‐related symptoms. We hypothesized that the single‐session, telehealth intervention would be feasible for patients with cancer pain, indicated by a moderate completion rate and high acceptability ratings.

## 2 Methods

### 2.1 Procedures

Patients with cancer pain were recruited from the community and from outpatient oncology clinics at the Stephenson Cancer Center (SCC) in Oklahoma City, Oklahoma between January and December 2023. Patients were eligible to participate if they were adults aged ≥ 18 years, endorsed cancer‐related pain (≥ 4/10 average pain intensity in the past 2 weeks), and were receiving active cancer treatment or completed treatment in the last 3 months. Patients without English fluency and/or who were cognitively impaired were excluded. To identify eligible participants, study staff screened outpatient hematology/oncology and supportive care clinics and received clinician referrals. Individuals identified by the study team were approached in person, eligibility was confirmed, and they provided written consent to participate. The class was also advertised via posters in SCC waiting rooms, new‐patient packets, and SCC's online calendar allowing individuals to self‐enroll using a web‐based consent form. Participants consented to attend a one‐time telehealth class, complete four electronic surveys, and to participate in an optional interview. Study procedures were approved by the University of Oklahoma Health Sciences Center Institutional Review Board (IRB #14680).

All study surveys were administered using REDCap [31, 32]. After consenting, participants were prompted to complete a baseline survey and intervention scheduling survey (offered monthly). Study staff emailed the Zoom link to participants and sent text message reminders before the intervention. Immediately following the intervention, participants received an emailed acceptability survey, along with links to three articles reiterating class content (pain‐CBT education principles, introduction to pain neuroscience, biopsychosocial aspects of pain, and evidence based coping skills) and three cancer pain specific audio‐relaxation exercises (cancer specific, pain‐CBT‐based relaxation exercises) [33]. Following attendance, participants were offered to participate in an optional qualitative, semi‐structured interview to evaluate facilitators and barriers to telehealth engagement and satisfaction with intervention delivery and content. Participants received a $50 gift card upon attendance and completion of the acceptability survey, and an additional $25 gift card for completing 2‐ and 4‐week follow‐up surveys.

#### 2.1.1 Cancer Pain 101 Intervention

**FIGURE 1:**
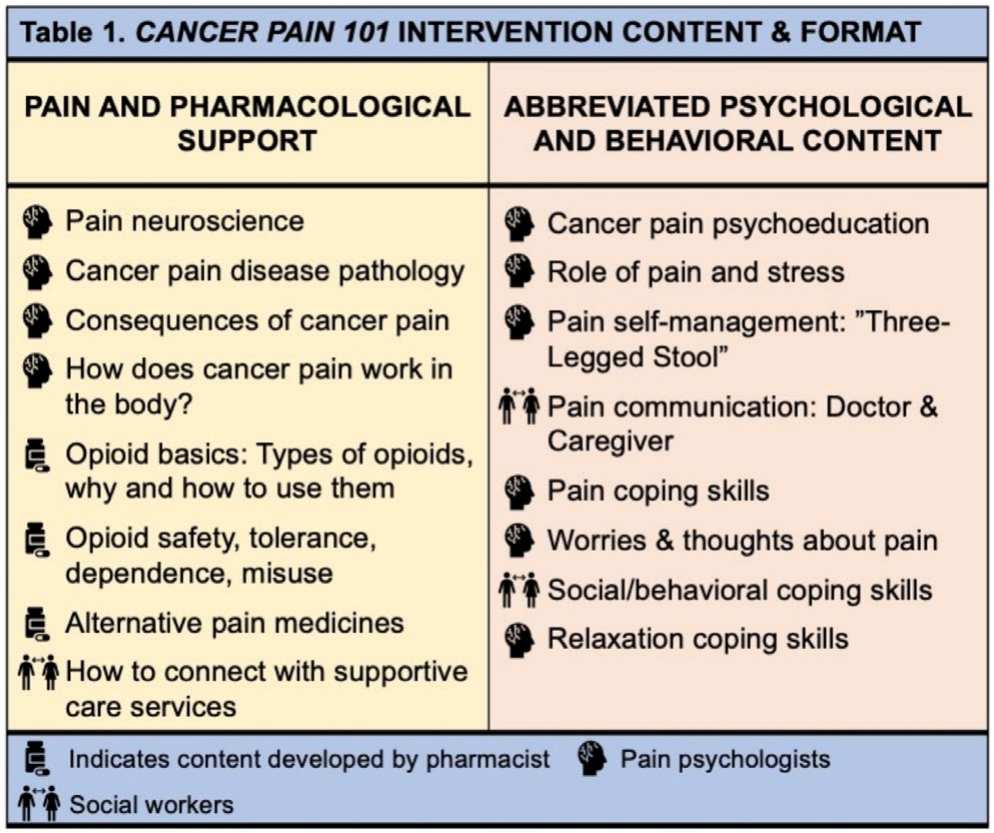
Topical summaries of Cancer Pain 101 content.

The 90‐min group intervention was hosted on Zoom. Intervention content was developed and presented by D.R.A. (pain psychologist) with feedback from J.H.S. (palliative pharmacist), and R.L. and K.B. (clinical social workers with experience delivering pain‐CBT). The course didactics focused on pain pharmacological support and pain‐CBT content for cancer pain self‐management. First, the pain psychologist introduced the biopsychosocial model of pain, basics of cancer pain disease pathology, and pain neuroscience. Then, the palliative pharmacist provided education on the evidence base for opioids, non‐opioids, and adjuvant medications, discussed accurate expectations for using opioids, defined tolerance, dependence, and misuse, and reviewed techniques for using opioids accurately and safely. Lastly, the licensed clinical social workers reviewed physical, behavioral, and psychological pain coping skills—including cancer‐specific examples and tips for improved pain communication (Figure 1). Finally, 15 min was allotted for questions.

### 2.2 Study Assessment and Measures

At baseline, participants completed study specific measures identifying socio‐demographic and clinical characteristics (age, gender, race, ethnicity, education, address, cancer type and stage, and previous cancer treatments), opioid use, and healthcare utilization (pain‐related calls to care team and emergency department visits). Immediately after the class, participants completed a post‐intervention acceptability survey. Two‐ and 4‐weeks following attendance, participants were emailed follow‐up surveys assessing clinical outcomes.

#### 2.2.1 Acceptability

Acceptability was assessed using adapted items from a self‐report acceptability survey [34], reviewing understandability, relevance, and usefulness of the content, and enjoyment of the class. Responses were rated on a 5‐point Likert scale (1–5) with higher scores indicating better acceptability. Study‐specific measures of acceptability were also included.

#### 2.2.2 Pain Intensity and Pain Interference

Pain severity and interference were measured using Patient‐Reported Outcomes Measurement Information System (PROMIS) [35]. Specifically, participants rated their average, worst, and least pain intensity in the last 7 days on the PROMIS‐Pain Intensity scale. All responses were measured on a modified scale—0 (no pain) to 10 (extreme pain). The pain intensity score was computed as the mean of the three pain intensity items, with higher scores indicating worse pain intensity. The PROMIS‐pain interference (6‐items) scale measured the extent to which pain interfered with participants' social, cognitive, emotional, physical, and recreational activities. All responses were measured on a 5‐point Likert scale: 1 (not at all) to 5 (very much) and the scale had excellent reliability (Cronbach's *α* = 0.96).

#### 2.2.3 Psychological Symptoms

Pain catastrophizing was measured using the Pain Catastrophizing Scale (PCS) [36], a 13‐item self‐report measure. Participants can rate their experience of maladaptive pain thoughts on a 5‐point Likert scale ranging from 0 (not at all) to 4 (all the time). Higher total scores indicate greater pain catastrophizing (range 0–52). The PCS had excellent internal consistency (Cronbach's *α* = 0.95). Self‐efficacy was measured using the 8‐item PROMIS Self‐Efficacy Questionnaire that targets patients' confidence in managing daily activities despite their health conditions on a 5‐point Likert scale and had excellent internal reliability (Cronbach's *α* = 0.89). Participants rated their sleep disturbance using the 4‐item Sleep Disturbance PROMIS scale [37] with scores reported on a 5‐point Likert scale: 1 (very good)–5 (very poor) with moderate internal reliability (Cronbach's *α* = 0.72). Depression symptoms were measured using the 4‐item PROMIS‐Depression questionnaire with scores ranging from 1 (never) to 5 (always) and had excellent internal reliability (Cronbach's *α* = 0.90). All PROMIS scale scores were summed and converted to a *t*‐score, with higher scores indicating greater symptomatology.

#### 2.2.4 Medication Use

Patients were asked about their use of prescribed opioids, non‐opioid prescribed pain, and mood medications. Surveys asked patients the frequency with which they were taking each medication. See Figure 4 for further details.

#### 2.2.5 Pain Coping

At baseline, 2‐week, and 4‐week follow‐up surveys, participants were asked to indicate pain coping strategies they currently use for cancer pain. See Table 4 for further details.

### 2.3 Statistical Analysis

Patient demographics, acceptability, medication use, and satisfaction ratings were evaluated using descriptive and frequency analyses. In line with previous reviews outlining recommendations for pilot trials [38] of health behavior interventions and the ORBIT model of intervention development [39], feasibility focused on participant attendance rates and acceptability of the intervention content. Feasibility was pre‐defined as ≥ 70% of participants attending the course and acceptable if ≥ 80% of the acceptability items were rated ≥ 4/5. While not powered to assess efficacy, we conducted paired sample *t*‐tests to explore within‐subject longitudinal changes between baseline and 2‐week follow‐up surveys and between baseline and 4‐week follow‐up surveys of self‐reported pain and psychological symptoms. Statistical analyses were conducted using SPSS version 26.0. Participants' self‐reported, open‐ended responses on the surveys were reviewed and summarized.

### 2.4 Qualitative Analysis

The interviewer listened to interview audio recordings and added quotes to interview notes, a procedure commonly used in rapid qualitative analysis [40, 41]. Later, a third study team member listened to the audio recordings, independently took notes, and compared them to the initial interview notes. Reviewing both written responses to free‐text survey questions and interview notes, we identified and summarized frequent pieces of patient feedback. Additionally, quotes were categorized to further illuminate key quantitative findings, such as feasibility and acceptability, as well as exploratory outcomes and preferences for intervention characteristics (e.g., interactivity, session length). Patients' comments that occurred infrequently but were emphasized were also noted.

## 3 Results

### 3.1 Demographics, Feasibility, and Acceptability Outcomes

**FIGURE 2:**
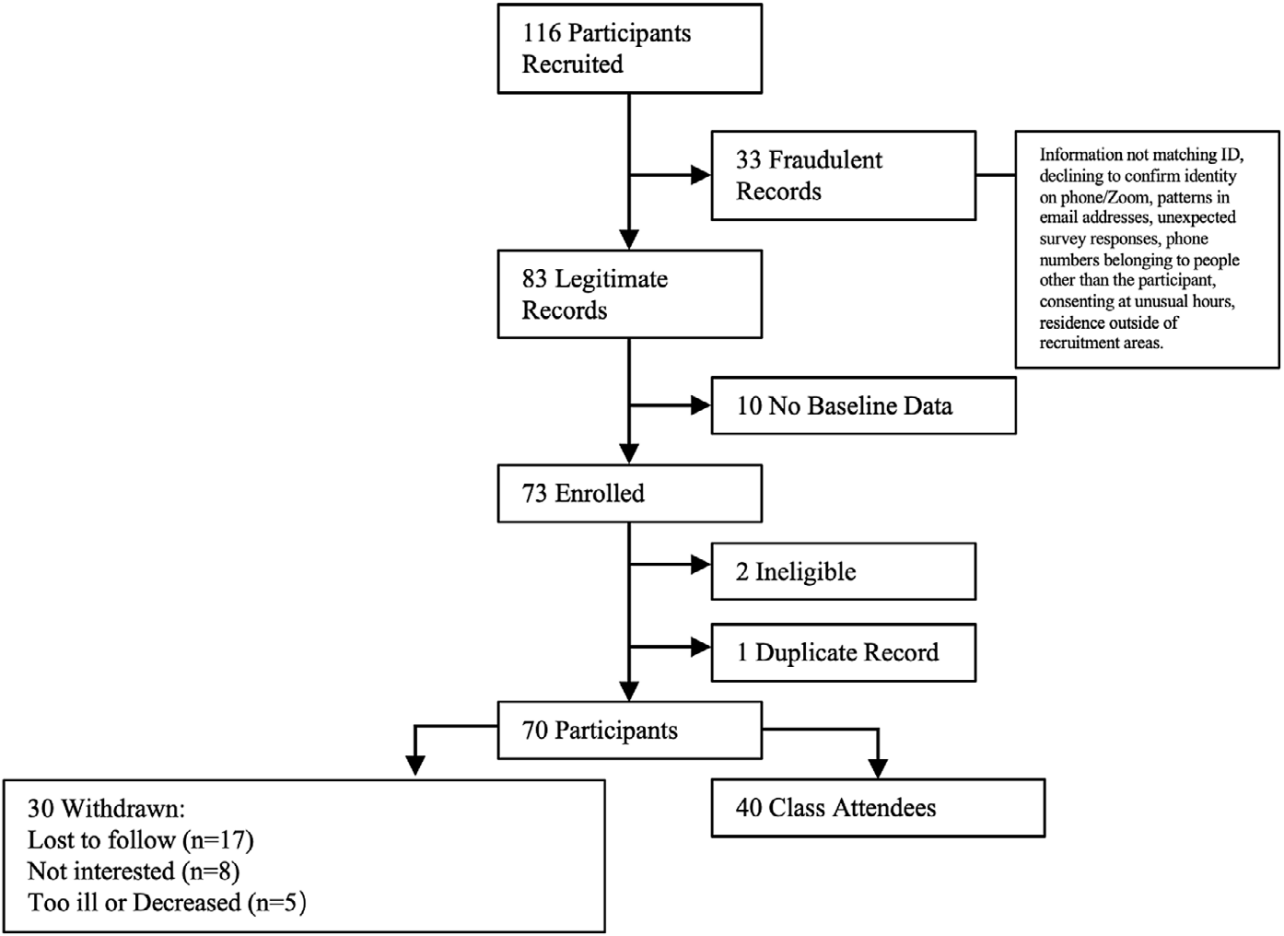
CONSORT diagram of uncontrolled pilot study of single‐session Cancer Pain 101 intervention.

Of the 166 patients who were recruited, 33 were identified as erroneous records and 10 without baseline data were removed. Among the 73 remaining patients, 2 were ineligible and 1 had a duplicate record. Out of the 70 enrolled participants, 30 were withdrawn for various reasons: lost to follow‐up (*n* = 17), no longer interested (*n* = 8), and too ill or deceased (*n* = 5). See Figure 2 for the CONSORT diagram. Of the 70 participants, 40 attended 9 of the separately offered intervention classes, resulting in a 57% attendance rate. Of those, 38 completed the post‐intervention survey (95%), 36 completed the 2‐week follow‐up survey (90%), and 37 completed the 4‐week follow‐up survey (92.5%). Of the 40 attendees, 5 engaged in the semi‐structured qualitative interview (12.5%) and 27 (67.5%) provided free text comments on the post‐intervention survey.

Among intervention attendees, more females (67.5%) participated, and participants were mostly white (87.5%), 52.5 (SD = 2.51) years of age on average, and diagnosed with various cancer types (e.g., gynecologic 27%, gastrointestinal 20%, and head/neck 11%, Table 1). Participants had various stages of cancer, but most had later stage cancers (stage 3/4 = 28, 70%). Ten participants (25.6%) lived in rural areas. At baseline, 28 (68%) participants reported taking opioids (short‐acting = 23, long‐acting = 15, both = 10) and 8 (20%) reported using cannabis for pain. Average pain intensity was rated as 6.1/10 (SD = 1.9). We compared demographics between attendees and non‐attendees (see Table 1). Non‐attendees differed significantly for race (*p* = 0.002) and educational attainment (*p* = 0.009). The seven black patients who enrolled in the study all withdrew or were lost to follow‐up (Table 1).

**TABLE 1:** Baseline sample characteristics.

Variables	Attended the class (*N* = 40)	Withdrew (*N* = 30)	*p*
Age [mean (SD)]^a^	52.51 (2.51)	52.7 (3.85)	0.481
Gender, *n* (%)			0.824
Female	27 (67.5)	21 (70)	
Male	13 (32.5)	9 (30)	
Race, *n* (%)^b^			0.012*
White	34 (85)	21 (70)	
Black	0 (0)	7 (23.3)	
Multiple	2 (5)	2 (6.7)	
Others	4 (10)	0 (0)	
Education, *n* (%)^b^			0.009*
Some high school level	2 (5)	5 (16.7)	
High school diploma	18 (45)	3 (10)	
Some college level (e.g., AA)	9 (22.5)	14 (46.7)	
College degree	6 (15)	5 (16.7)	
Higher level degree (e.g., MA, MS, PhD)	5 (12.5)	3 (10)	
Cancer type, *n* (%)^b^			0.671
Gynecologic	12 (30)	6 (20)	
Gastrointestinal	6 (15)	6 (20)	
Lung	4 (10)	3 (10)	
Head/neck	2 (5)	4 (13.3)	
Others	16 (40)	11 (36.7)	
Cancer stage, *n* (%)^b^			0.128
I	6 (16.2)	2 (7.7)	
II	3 (8.1)	5 (19.2)	
III	5 (13.5)	8 (30.8)	
IV	23 (62.2)	11 (42.3)	
Rural or urban, *n* (%)^b^			0.872
Rural	10 (25)	7 (23.3)	
Urban	30 (75)	23 (76.6)	
In the past 12 months, was there ever a time when you needed to see a medical specialist about a health issue but did not get it, *n* (%)^b^			0.281
Yes	6 (15)	7 (23.3)	
No	34 (85)	23 (76.7)	
Number of times called care team members for help with cancer pain in the past 12 months, *n* (%)^b^			0.310
0	8 (20.0)	7 (23.3)	
1–3	23 (57.5)	12 (40.0)	
4 or more	9 (22.5)	11 (36.7)	
Number of times visited the emergency department for pain in the past 12 months, *n* (%)^b^			0.704
0	13 (32.5)	12 (40.0)	
1–3	24 (60.0)	15 (50.0)	
4 or more	3 (7.5)	3 (10.0)	
Shorting‐acting opioids use at baseline, *n* (%)^b^			0.094
Yes	23 (57.5)	23 (76.6)	
No	17 (42.5)	7 (23.3)	
Long‐acting opioids use at baseline, *n* (%)^b^			0.111
Yes	15 (37.5)	17 (56.6)	
No	25 (62.5)	13 (43.3)	
Baseline pain intensity [mean (SD)]^a^	6.10 (1.90)	6.14 (2.13)	0.938

^a^ Independent *t*‐test was used for continuous variables.

^b^ Chi‐squared test was used for categorical variables, Fisher's exact test was used when observed variables had < 5.

* *p* ≤ 0.05.

Of attendees who completed the acceptability measures (*n* = 39/40), the majority of participants reported that the content was understandable (97%, *n* = 38), relevant (90%, *n* = 35), and useful (82%, *n* = 32), and that they were likely to use the skills they learned from the intervention (82%, *n* = 32). In total, 78% rated their overall satisfaction with the class as ≥ 4/5 (*n* = 31/40). Most reported that they enjoyed attending (77%, *n* = 30) and learned from the content (74%, *n* = 29). Many participants reported that the intervention was helpful for pain self‐management (69%, *n* = 27) and that it was easy to connect to via Zoom (72%, *n* = 28); only two (5%) reported that it was not easy to connect. In total, the majority of participants (78%, *n* = 31) said they would recommend *Cancer Pain 101* to a friend (Table 2).

**TABLE 2:** Acceptability ratings of Cancer Pain 101 intervention from attendees.

Item	4–5/5 Likert scale, % (*n*)
How understandable was the content in the Cancer Pain 101 class?^a^	97 (38)
How relevant was the content presented during the Cancer Pain 101 class?	90 (35)
How much did you enjoy attending the Cancer Pain 101 class?^a^	77 (30)
How much did you learn from the Cancer Pain 101 class about pain management?	74 (29)
How useful was the information presented in the Cancer Pain 101 class?	82 (32)
How likely are you to use the skills and information you learned in the class?	82 (32)
How helpful was the Cancer Pain 101 class for your pain management?^a^	69 (27)
How easy was it to connect to the Cancer Pain 101 class via Zoom?^a^	72 (28)
How would you rate your overall satisfaction with the Cancer Pain 101 class?^a^	80 (31)
How likely would you be to recommend the Cancer Pain 101 class to another patient?	80 (31)

*Note:* All responses are scored on a 5‐point Likert Scale 1–5, with higher scores representing higher acceptability.

^a^ Indicates modified items for the Acceptability E‐scale.

Attendees expressed positive feedback about the class. One participant said, “this class [gave] me some tools to better manage my pain … and tools to be able to find help if I need it” (Female, 65 years old, stage III cervical cancer). Another attendee (Female, 79 years old, Stage II breast cancer) stated, “The class presented new information regarding pain management strategies and the types of pain cancer can cause.” Yet one attendee (Female, 40 years old, Stage IV ovarian cancer) described that the information duplicated what she learned from palliative care, “For somebody that's new … I feel like it's good information. For me, it was kind of hard. I feel like I just kind of already knew because I've done the palliative care, so I kind of knew a lot of it,” and emphasized the class may be best for patients early in their treatment trajectories.

The intervention duration was also well‐received: “It wasn't too long, wasn't too short. I think all the questions were answered that needed to be” (Female, 79 years old, Stage II breast cancer). The convenience of attending virtually was highlighted. For example, one participant (Male, 54 years old, Stage IV bladder cancer) mentioned, “The format was very good. Having the three speakers helped keep your attention focused.” Another stated, “I'm all about telehealth … It just makes it so much easier and more convenient,” (Female, 42 years old, Stage I ovarian cancer). Yet one participant pointed out, “Maybe more interaction with those attending … I got distracted a lot because it was just information given to me.” (Female, 40 years old, Stage IV ovarian cancer).

### 3.2 Exploratory Treatment Outcomes

**FIGURE 3:**
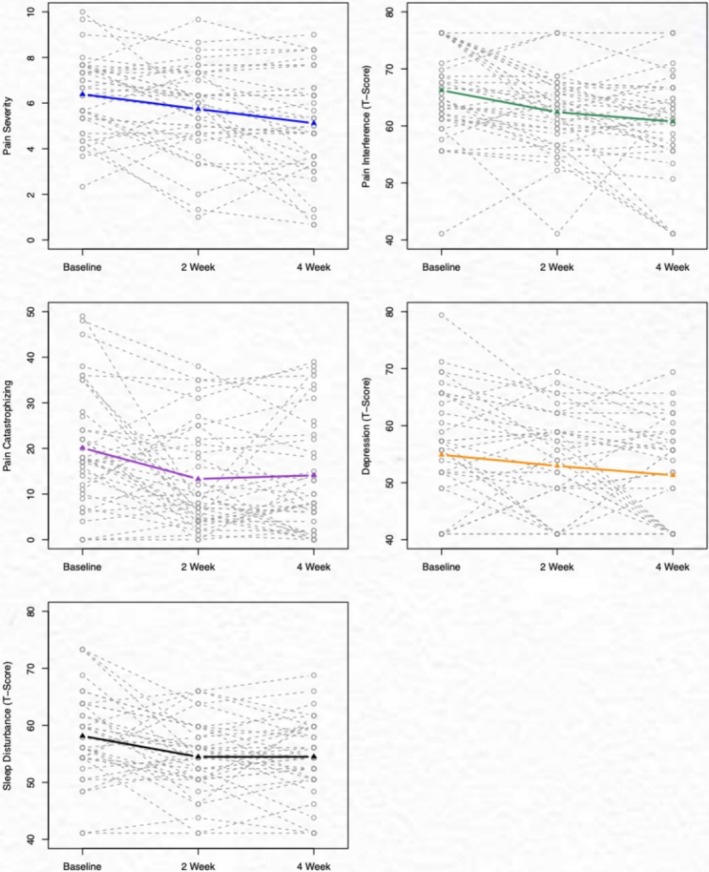
Changes in pain intensity, pain interference, and pain catastrophizing from baseline to 2–4 week follow‐up. Line graphs display participants' changes in pain and other related symptoms measured at baseline, 2 weeks, and 4 weeks. Each line with open circles and connected with dash represents an individual participant's trajectory over the three time points. Solid lines indicate the mean trajectory, showing the general trends in symptoms over time.

Pain intensity significantly decreased from baseline to follow‐up at 2 and 4 weeks (mean difference [MD] = 0.65, *p* = 0.02; MD = 1.27, *p* = 0.001 [mean difference calculated with baseline—2 week follow‐up or baseline—4 week follow‐up]), respectively. Pain interference and pain catastrophizing also decreased significantly from baseline to the 2‐week follow‐up (MD = 3.85, *p* < 0.001; MD = 6.60, *p* = 0.004) and to the 4‐week follow‐up (MD = 5.48, *p* = 0.017; MD = 6, *p* = 0.003). Sleep disturbance and depressive symptoms improved from baseline to the 2‐week follow‐up (MD = 3.76, *p* = 0.002; MD = 2.33, *p* = 0.080) and the 4‐week follow‐up after the intervention (MD = 3.64, *p* = 0.004; MD = 3.97, *p* = 0.018). There was no change in pain self‐efficacy (Table 3; Figure 3).

**TABLE 3:** Changes in pain and other symptoms from baseline to 2‐week and baseline to 4‐week follow‐up.

Variable	Time point	Mean (SD)	*p*
Pain intensity (average) (0–10)	Baseline	6.39 (1.70)	
	2 weeks	5.74 (1.97)	0.020*
	4 weeks	5.12 (2.27)	0.001*
Pain interference (*t* score: 0–100)	Baseline	66.26 (1.37)	
	2 weeks	62.41 (1.19)	0.001*
	4 weeks	60.78 (1.52)	0.017*
Pain catastrophizing (0–52)	Baseline	20.11 (13.21)	
	2 weeks	13.31 (11.33)	0.004*
	4 weeks	14.11 (12.75)	0.003*
Self‐efficacy in managing symptoms (*t* score: 0–100)	Baseline	50.00 (10.00)	
	2 weeks	50.01 (10.01)	0.901
	4 weeks	50.00 (10.00)	> 0.999
Sleep disturbance (*t* score: 0–100)	Baseline	58.24 (7.78)	
	2 weeks	54.48 (6.46)	0.002*
	4 weeks	54.60 (6.74)	0.004*
Depression (*t* score: 0–100)	Baseline	55.26 (10.63)	
	2 weeks	52.93 (9.14)	0.080
	4 weeks	51.29 (9.06)	0.018*

*Note:* *p* values reference changes in scores compared to baseline using paired sample *t*‐test. Measures: Pain intensity: PROMIS Pain Intensity 3‐item Scale = average score; Pain interference: PROMIS Pain Interference 6‐item Scale; pain catastrophizing: 13‐item Pain Catastrophizing Scale; Self‐efficacy: PROMIS Self‐Efficacy for Managing Symptoms – Short Form 8a; Sleep Disturbance: PROMIS Sleep Disturbance 4‐item Scale; Depression: PROMIS Depression 4‐item Scale. *p < 0.05.

Through interviews and survey notes, participants shared how the intervention helped them manage their pain symptoms. By gaining a comprehensive understanding of cancer pain, participants described feeling less “helpless” about their pain, with one participant stating, “I feel this class gave me some tools to better manage my pain. That I am not helpless and alone, and tools to be able to find help if I need it. Pain is a part of my life now, but it doesn't have to control my life. Thanks!” (Female, 65 years old, stage I cervical cancer).

Two participants described that the intervention helped them address their reluctance to use pain medications due to addiction concerns. The intervention encouraged them to have productive discussions with their doctors. One stated,

You hear so much about all these people that get addicted to pain pills … You know, I don't feel I'm addicted to them, but I tell you what, I now set my alarm to take them. So I take them and it makes a world of difference … I thought the class helped me with that, and like I said, it spurred me on to have the talk with my doctor. (Female, 69 years old, stage IV uterine cancer)

Another described:

It really helped. I was nervous to tell [my doctor]. I didn't want anyone to act like I was just out looking for more pain medicine … Just you guys telling me … that it was okay for me to talk to my doctor, tell them that my pain medicine wasn't working … This really helped me with that. (Female, 28 years old, stage IV colorectal cancer)

#### 3.2.1 Changes in Pain Management Coping Skills

**FIGURE 4:**
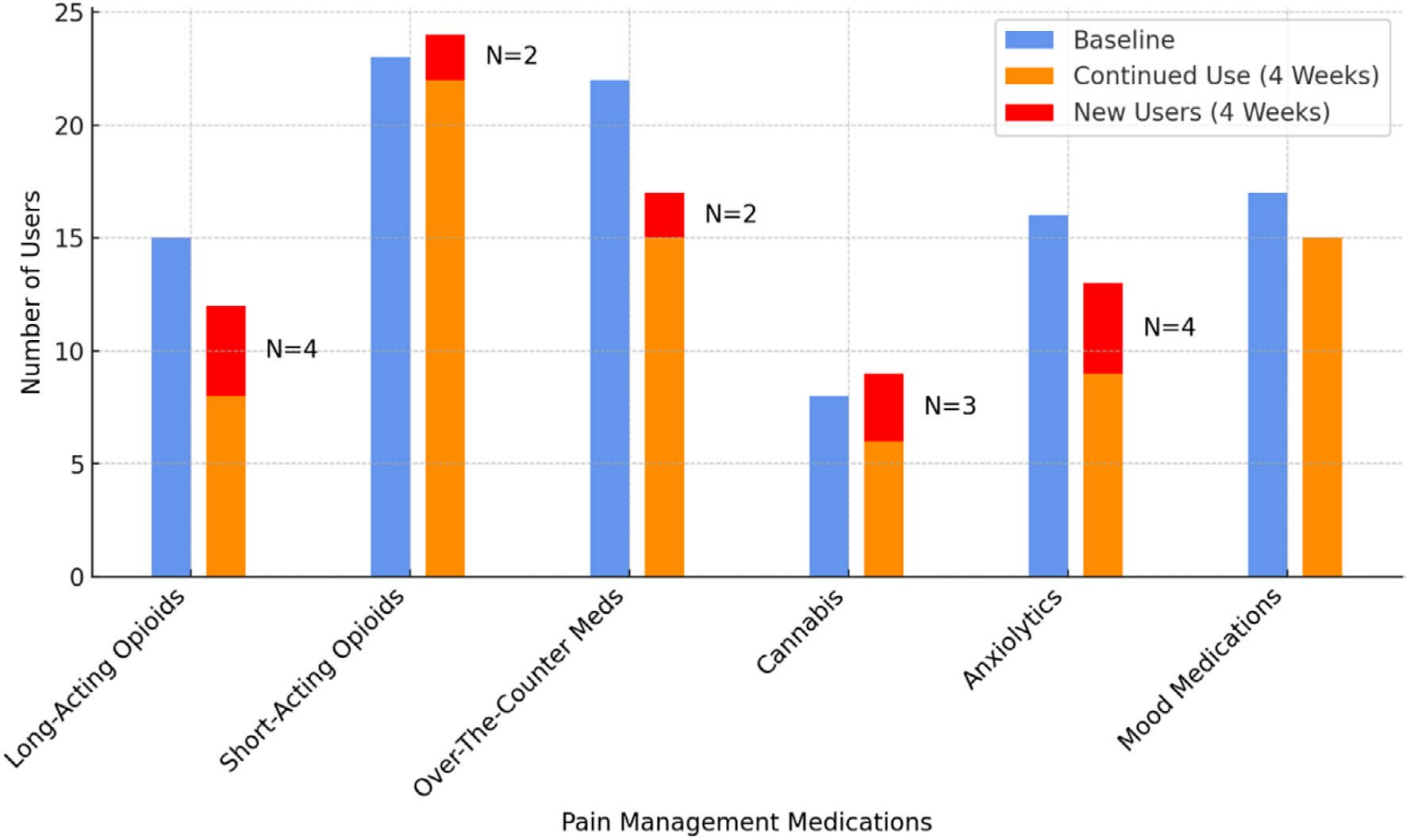
Pain management medications change from baseline to 4 weeks. Patients were asked about their use of prescribed opioids, non‐opioid prescribed pain medications, over‐the‐counter pain medications, prescribed mood, sleep, and anxiolytic medications, and cannabis use during baseline and 4‐week follow‐up surveys. Items were phrased as “Are you taking [short‐acting/breakthrough opioid pain medicine(s) (usually prescribed to take as needed every 4–6 h)?] response options ‘yes/no’” for each medication type. Prescription for short‐ or long‐acting opioids were also abstracted from electronic medical records after class attendance.

Several participants reported initiating pain medications following the intervention, including opioids and over‐the‐counter medications (Figure 4). Several reported they increased the use of nonpharmacological pain management techniques including meditation/relaxation, yoga/stretching, massage, and movement/physical activity (Table 4). Meditation and relaxation were directly encouraged during the intervention and links to cancer pain‐specific audio‐recorded relaxations were sent to attendees following class completion. One participant shared that she initiated new skills following the class, “Some of the things I hadn't thought about before … like the meditation stuff and just the different breathing exercises.” (Female, 40 years old, Stage IV ovarian cancer).

**TABLE 4:** Changes in self‐reported use of pain coping strategies.

Pain coping strategies	Baseline (*n* = 40)	4‐Weeks follow up (*n* = 37)
	*N* (%)	*N* (%)
Massage	**7 (17.5)**	**10 (27)**
Head or cold packs/hot baths	28 (70)	25 (67.6)
Movement/physical activity	**14 (35)**	**18 (48.6)**
Counseling/behavioral therapy	7 (17.5)	6 (16.2)
Spending time with others	10 (25)	11 (29.7)
Acupuncture/chiropractic	0 (0)	0 (0)
Yoga or stretching	**4 (10)**	**9 (24.3)**
Meditation or relaxation	**5 (12.5)**	**13 (35.1)**
Prayer or religious activities	12 (30)	12 (32.4)
Other^a^	3 (10)	4 (10.8)

*Note:* Patients were queried about pain management strategies at baseline and at 4‐week follow‐up “Which of the following do you currently use to cope with cancer pain? Please select all appropriate options” with the responses listed above. ^a^At baseline, three patients indicated medication, and doing nothing. At 4‐week follow‐up, four patients mentioned medication, art, and doing nothing.

## 4 Discussion

Many patients with cancer experience inadequate pain control due to limited access to both comprehensive pain education that emphasizes pain self‐management techniques and alternative behavioral pain therapies. This pilot study tested the feasibility and acceptability of an integrated single‐session telehealth intervention that delivered comprehensive medical and psycho‐behavioral education about cancer pain, equipping participants with evidence‐based information about cancer pain self‐management strategies in a brief, accessible format. We observed a moderate completion rate (57%, 40 attendees/73 enrolled) that did not meet our pre‐set feasibility benchmark; However, high assessment completion rates at ≥ 90%, and high acceptability ratings (≥ 80% scores 4/5) were reported in questionnaires and described in qualitative feedback. While we were underpowered to definitively evaluate treatment outcomes, exploratory analyses revealed that participants reported significant reductions in pain intensity (clinically relevant reduction at 4 weeks), pain interference, pain catastrophizing, sleep disturbance, and depression over a 4‐week post‐intervention period. Incorporation of qualitative data from interviews and open‐text responses allowed for deeper insight into patient cancer pain experiences as many participants reported appreciating the pain management information received during the intervention.

While previous meta‐analyses have confirmed the positive impact telemedicine interventions can have on patients' pain outcomes [23], this intervention is particularly novel as it integrates clinical pain neuroscience and pharmacology education with behavioral pain coping strategies tailored for cancer pain together into a single‐session intervention. Behavioral pain management is often absent or inaccessible to patients with cancer. This single‐session intervention delivered pain and opioid education paired alongside introductions to evidence‐based pain coping skills (cognitive restructuring) with direct links to relaxation pain management recordings. Post‐intervention data revealed a substantial increase in the use of meditation and relaxation pain self‐management strategies that have been shown to alleviate cancer‐related pain and improve overall well‐being [42, 43]. Qualitative feedback demonstrated that Cancer Pain 101 provided some participants with knowledge and coping skills (cognitive restructuring) that resulted in reduced pain catastrophizing or feelings of helplessness and fear associated with their pain; pain catastrophizing has frequently been cited as being significantly associated with worse pain outcomes and greater opioid delivery in patients with cancer [44, 45]. The opioid education within the intervention provided patients with critical information about the safe use of opioids and promoted the integration of alternative pain self‐management strategies [19, 46]. Hesitance to use opioids has been increasingly identified among patients with cancer, resulting in uncontrolled pain [47, 48]. While a considerable number of participants reported using traditional pharmacological solutions before and after the intervention, participants described that this intervention mitigated concerns about addiction and thwarted discussions about opioids with their doctors. While most patients found the intervention informative, unsurprisingly, prior engagement with palliative care reduced the novelty of the information. Thus, delivery within outpatient oncology care, either before palliative care referral or within patients who will not receive palliative care, would potentially be more useful.

Importantly, this single‐session telehealth design is built on previous models [49, 50] that overcome challenges associated with in‐person or multi‐session interventions, such as clinician burden or transportation barriers [51, 52]. The intervention successfully minimized technical barriers using a user‐friendly telehealth interface. Patients who attended rarely voiced concerns with Zoom delivery, and 25% of the study sample lived in rural areas in Oklahoma—a population who has limited access to comprehensive pain therapies. Notably, the pre‐set feasibility benchmark of 70% attendance was not met, indicating that some patients may still be limited by a discomfort with technology, being too sick, or having a lack of interest. Extending positive findings of telemedicine interventions for cancer pain [23], this single‐session intervention can serve as a vital and *brief* introduction to cancer pain self‐management skills for patients with cancer.

Future Cancer Pain 101 refinements may include integrating delivery of the intervention within oncology, thus ensuring novelty and timeliness of these skills by providing access to patients near their diagnosis and streamlining pain services that can reduce clinician and patient burden. Cancer Pain 101 refinements may also include a more customized approach, assessing participants' prior knowledge and adjusting content accordingly [53]. Patients requiring more intensive pain management could be referred for further specialized outpatient treatment or may benefit from increased monitoring. As identified in a similar meta‐analysis, telemedicine for cancer pain can be effective and further research is needed to determine the optimal timing to deliver pain self‐management content that incorporates telehealth into existing healthcare systems [23]. Although we were unable to capture reasons why patients were lost to follow‐up, we are conducting a randomized feasibility study that may be able to better qualify barriers and modify delivery of the intervention accordingly. To reduce the rate of fraudulent enrollments, we plan to increase in‐person clinical recruitment and integrate a checklist to confirm online participant identities [54].

The uncontrolled, single‐arm pilot study format restricts our ability to establish a causal relationship between the intervention and observed changes in pain outcomes. Unfortunately, many participants maintained elevated average pain severity > 5/10 at the end of the study, paralleling findings that patients with cancer continue to experience significant pain even while receiving opioid therapy [5, 6]. While the study was not pre‐registered as a clinical trial, it provides evidence for the feasibility and acceptability of a single‐session intervention to be tested in a larger efficacy study, as outlined in the ORBIT model for intervention development [39]. The small sample size and lack of sample diversity—primarily white‐identifying individuals with advanced cancer—limit the generalizability of study findings. Also, while sample sizes were small, more Black patients withdrew from the study and did not respond to calls to engage in qualitative interviews. To ensure the intervention is equally accessible and beneficial to all patients with cancer pain, continued qualitative exploration is necessary to address and reduce demographic differences in uptake.

## 5 Conclusion

Over half of participants attended and reported high acceptability of our Cancer Pain 101 intervention, a single‐session, telehealth, interdisciplinary cancer pain education class that combined psychological and pharmacologic content to improve cancer pain self‐management. Exploratory analyses suggest important short‐term, post‐intervention reductions in pain intensity, interference, sleep disturbance, and depression. The intervention utilized telehealth technology to offer a single‐session, scalable, convenient pain management education tailored to the needs of patients with cancer pain that integrated with their current oncologic care. Future feasibility and efficacy testing is needed to ensure integration of Cancer Pain 101 in cancer care delivery and to comprehensively evaluate its potential clinical impact.

## Author Contributions

**Desiree R. Azizoddin:** conceptualization (lead), data curation (equal), formal analysis (supporting), funding acquisition (lead), investigation (lead), methodology (lead), project administration (lead), supervision (lead), visualization (supporting), writing – original draft (lead), writing – review and editing (lead). **Jian Zhao:** formal analysis (equal), visualization (equal), writing – original draft (equal), writing – review and editing (equal). **Sara M. DeForge:** data curation (supporting), investigation (supporting), writing – original draft (supporting), writing – review and editing (supporting). **Meng Chen:** data curation (lead), formal analysis (lead), methodology (equal), writing – review and editing (equal). **Ryan Nipp:** methodology (supporting), writing – review and editing (supporting). **Jennifer Hardcopf Stagg:** investigation (equal), writing – review and editing (equal). **Kerry Bond:** investigation (equal), writing – review and editing (equal). **Raina Leckie:** investigation (equal), writing – review and editing (equal). **Blake T. Hilton:** writing – review and editing (equal). **Jordan M. Neil:** writing – review and editing (equal). **James A. Tulsky:** writing – review and editing (equal). **William Pirl:** writing – review and editing (equal). **Robert R. Edwards:** conceptualization (equal), methodology (equal), writing – review and editing (equal). **Beth D. Darnall:** conceptualization (equal), writing – review and editing (equal).

## Ethics Statement

The study was approved by the University of Oklahoma Health Sciences Center IRB (#14680).

## Consent

All the patients provided informed consent to participate.

## Conflicts of Interest

The authors declare no conflicts of interest.

## Data Availability

The data that support the findings of this study are available upon request.
